# Effects of Addition of Preoperative Intravenous Ibuprofen to Pregabalin on Postoperative Pain in Posterior Lumbar Interbody Fusion Surgery

**DOI:** 10.1155/2017/1030491

**Published:** 2017-08-16

**Authors:** Hüseyin Ulaş Pınar, Ömer Karaca, Fatma Karakoç, Rafi Doğan

**Affiliations:** Baskent University Konya Research Center Anesthesiology Department, Hocacihan Mah. Saray Cad. No. 1, Selçuklu, Konya, Turkey

## Abstract

**Objective:**

Ibuprofen and pregabalin both have independent positive effects on postoperative pain. The aim of the study is researching effect of 800 mg i.v. ibuprofen in addition to preoperative single dose pregabalin on postoperative analgesia and morphine consumption in posterior lumbar interbody fusion surgery.

**Materials and Methods:**

42 adult ASA I-II physical status patients received 150 mg oral pregabalin 1 hour before surgery. Patients received either 250 ml saline with 800 mg i.v. ibuprofen or saline without ibuprofen 30 minutes prior to the surgery. Postoperative analgesia was obtained by morphine patient controlled analgesia (PCA) and 1 g i.v. paracetamol every six hours. PCA morphine consumption was recorded and postoperative pain was evaluated by Visual Analog Scale (VAS) in postoperative recovery room, at the 1st, 2nd, 4th, 8th, 12th, 24th, 36th, and 48th hours.

**Results:**

Postoperative pain was significantly lower in ibuprofen group in recovery room, at the 1st, 2nd, 36th, and 48th hours. Total morphine consumption was lower in ibuprofen group at the 2nd, 4th, 8th, 12th, and 48th hours.

**Conclusions:**

Multimodal analgesia with preoperative ibuprofen added to preoperative pregabalin safely decreases postoperative pain and total morphine consumption in patients having posterior lumbar interbody fusion surgery, without increasing incidences of bleeding or other side effects.

## 1. Introduction

Acute postoperative pain remains to be a major problem, and inadequate or excessive treatment can cause side effects, including increased risk of myocardial ischemia or infarction, thromboembolic and pulmonary complications, persistent postoperative pain incidence, alterations in immune system, impaired rehabilitation, increased hospital stay or readmissions to the hospital, impaired quality of life, and excessive sedation due to opioid use [1]. There are developments regarding novel analgesic medical treatments and techniques to be employed in preoperative, intraoperative, and postoperative periods to better manage acute postoperative pain. Approaches targeting preoperative period are increasing in prevalence and are referred to as preventive analgesia. Preventive analgesia aims to decrease postoperative pain with ongoing analgesic effect during surgery, therefore decreasing afferent nociceptive neurotransmission caused by surgical stimulation [2].

Patients having lumbar spinal fusion or laminectomy surgeries often complain about severe postoperative pain and postoperative rehabilitation process can be effected negatively [3, 4]. Many methods have been tested for preventive analgesia in these types of surgeries. These methods include neuroaxial opioid administration like continuous subcutaneous morphine [3], intrathecal morphine injection [3], epidural morphine [4], caudal bupivacaine and tramadol administration [5], diclofenac sodium [6], and i.v. administration of dexketoprofen and paracetamol [7].

Although pregabalin was approved as antiepileptic and for chronic pain, because of its antinociceptive properties it is also used for perioperative pain management. Clinical studies related to perioperative pain indicate that it has effects on reducing acute neuropathic pain and decreasing opioid dose [8]. Administration of a single dose of pregabalin before lumbar spinal surgery has been shown to reduce severity of postoperative pain and morphine consumption [9].

There are positive results related to preventive use of NSAIDs before lumbar surgery [10, 11]. The propionic acid derivative ibuprofen has analgesic and anti-inflammatory properties through central and peripheral blockade of cox-1 and cox-2 isoenzymes and can also have an effect through cox- independent pathways [12–14]. Intravenous ibuprofen has become available in 2009 and can be used for preemptive analgesia. Studies carried out in orthopedic surgery have shown preoperative and postoperative administration of i.v. ibuprofen reduced postoperative pain and opioid need [15]. We did not encounter any studies related to combined use of ibuprofen and pregabalin before lumbar spinal surgery.

We aimed to examine the effects of adding i.v. ibuprofen to preoperative single dose pregabalin on postoperative pain levels, morphine consumption, and side effects in surgical situations where postoperative pain is severe such as posterior interbody fusion surgery.

## 2. Materials and Methods

**Figure 1:**
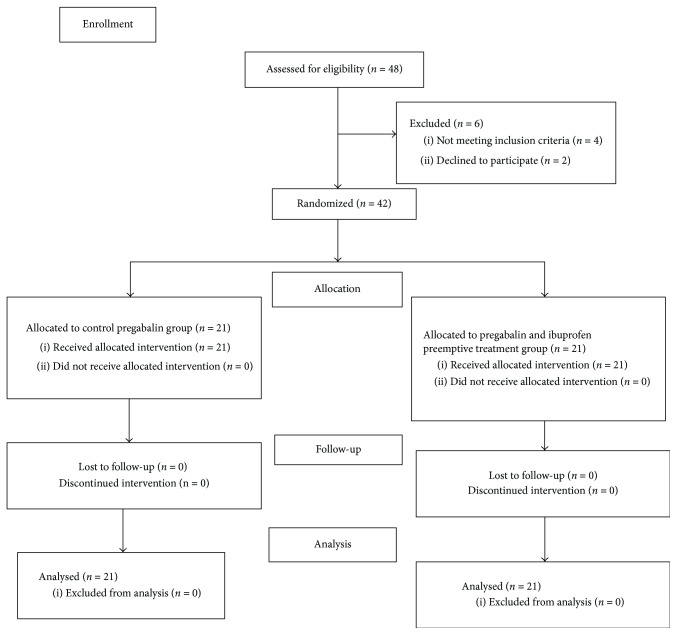
CONSORT flow diagram.

We included consecutive 42 elective lumbar stabilization patients between 18 and 65 years of age and with ASA (American Society of Anesthesiologists) I-II physical status. All participants were informed both verbally and in writing about details of the study before it started and informed consent was obtained from all participants. Approval from Baskent University Clinical Studies Ethical Committee was obtained (Chairperson Hakan Özkardeşler, Project number KA16/71). The study was registered to the Australian New Zealand Clinical Trial Registry (Number ACTRN12616000591459). We followed the Consolidated Standards of Reporting Trials (CONSORT) guidelines for reporting randomized trials and provided a CONSORT flow diagram (Figure 1).

Patients with history of antiepileptic drug use, allergy to drugs to be used, severe liver, renal and gastrointestinal system illnesses, and psychiatric illnesses, patients who refused to participate in the study, patients with long term opioid or NSAID use, pregnant or lactating patients, patients with diabetes or other neuropathic diseases, and patients who refused to use patient controlled analgesia (PCA) device were excluded.

Demographic data of the patients were recorded. Patients were divided randomly into two groups by computer program to have 21 patients in each group. All patients in both groups were given 150 mg pregabalin capsule (Lyrica, Pfizer, Germany) with a little water 1 hour prior to the surgery. Patients in Group PI (Group Pregabalin-Ibuprofen) received 800 mg i.v. ibuprofen (İntrafen, 800 mg/8 ml i.v., Gen İlaç, Turkey) 30 minutes before surgery in 250 ml saline infused over 30 minutes. Patients in Group P (Group Pregabalin) received 250 ml saline without ibuprofen. Patients were blinded to the study group. After patients were admitted to the operating room standard monitorization with electrocardiography (ECG), peripheral oxygen saturation (SpO_2_), and noninvasive blood pressure (NIBP) was implemented. Anesthesia induction was obtained with 0.02 mg/kg i.v. midazolam, 2 microgram/kg fentanyl, 2 mg/kg propofol, and 0.6 mg/kg rocuronium. Following endotracheal intubation patients were placed under prone position for operation and surgery was performed under inhalational anesthesia with 1-2% sevoflurane in 40%/60% O_2_/N_2_O. Type of operation, operated lumbar area, duration of the operation, intravenous fluids given during surgery, amount of hemorrhage, and amount of blood transfusion were recorded. Patients were transported to the postanesthesia care unit (PACU) after surgery following extubation.

All patients received 0.05 mg/kg morphine loading dose in PACU followed by morphine PCA (1 mg/ml, total 100 mg = 100 ml, bolus 1 mg, 10-minute lock interval, and 4-hour limit: 30 mg morphine) device. Intravenous 1 g paracetamol was administered every six hours, first dose to be administered upon arrival to the ward. Postoperative pain was recorded at 0, 1, 2, 4, 8, 12, 24, 36, 48 hours during resting as VAS value (VAS 0 cm = no pain; 10 cm = most severe pain). Pain level during ambulation at the 24th hour and patient satisfaction at the 48th hour were also evaluated by using VAS. Presence of side effects such as sedation, nausea, vomiting, dizziness, and visual disturbances was evaluated at the 1st, 2nd, 4th, 8th, 12th, 24th, 36th, and 48th hours (0 = absent; 1 = present). All of these postoperative evaluations were done by a researcher blinded to the patients' groups. Morphine consumption in the PCA was recorded simultaneously with VAS assessments.

To determine number of patients for the study 80% power and the difference of 1.5 units in VAS measurements were used. It was calculated that it would be appropriate to take 21 individuals in each group (total of 42 individuals). The difference of 1.5 units in VAS was determined by pilot study and clinical experience.

For testing categorical parameters between two groups, cross-tab analysis was used with Chi-square test, and for normally distributed continuous variables two independent groups' *t*-test or for nonnormal variables nonparametric two-independent sample Mann–Whitney *U* test was used. Repeated measurements were analysed by nonparametric *k*-related samples Friedman test with Chi-square test. All analyses were computed by using SPSS 15.0 statistical package program. *P* < 0.05 was accepted as significant.

## 3. Results

**Figure 2:**
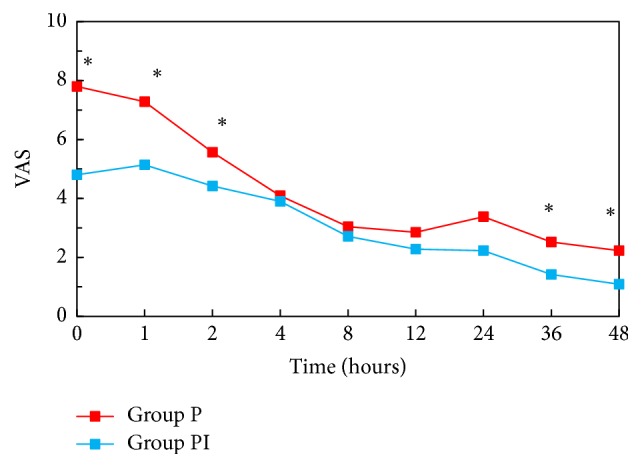
Postoperative pain scores. ^*∗*^Statistifical significance between two groups.

**Figure 3:**
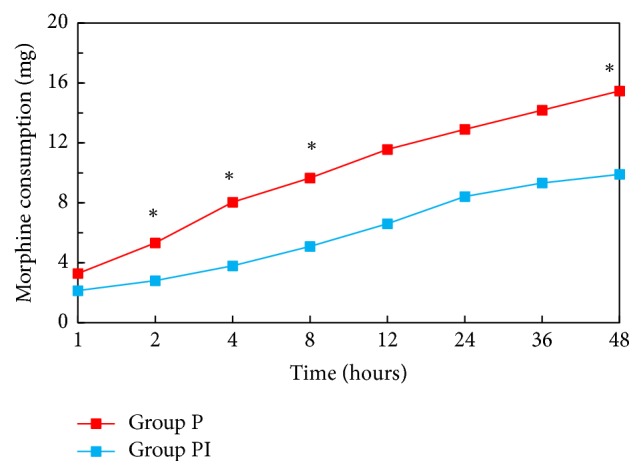
Total morphine consumption. ^*∗*^Statistifical significance between two groups.

Total of 42 patients, 21 patients in each group, were enrolled in the study between March 2016 and September 2016. Figure 1 illustrates the flow of the patients through the trial. Patient characteristics did not differ among two groups (Table 1). There was also no statistically significant difference between groups regarding number of operated lumbar regions, duration of surgery, hemorrhage during surgery, blood transfusion, and amount of fluids given during surgery (Table 2). VAS scores in Group PI were significantly lower at the 0th, 1st, and 2nd hours compared to the control group (*P* < 0.001, *P* = 0.002, and *P* = 0.047, resp.). Also at the 36th and 48th hours even though VAS scores were very low they were significantly lower in Group PI (Figure 2). VAS scores during ambulation evaluated at the 24th hour were significantly lower in Group PI when compared with Group P (Group PI: VAS = 3.28; Group P: VAS = 5.09; *P* = 0.05). Morphine consumption in Group P was significantly higher at the postoperative 2nd, 4th, 8th, 12th, and 48th hours (*P* = 0.002, *P* = 0.006, *P* = 0.009, *P* = 0.018, and *P* = 0.043, resp.) (Figure 3). There was no statistically significant difference between groups regarding patient satisfaction.

There was no significant difference between groups regarding sedation, nausea, vomiting, dizziness, and visual disturbances (Table 3).

## 4. Discussion

According to the results of this study preoperative single dose i.v. ibuprofen in addition to preemptive pregabalin significantly reduced pain levels in the first two postoperative hours and reduced morphine consumption especially in the first 12 hours. In a study with a total of 206 orthopedic or abdominal surgery patients, postoperative pain levels and morphine consumption were reported to be lower in patients who received 800 mg i.v. ibuprofen every 6 hours [15]. Again in another study with 406 abdominal or orthopedic surgery patients, placebo and 400 mg and 800 mg of ibuprofen were compared and it has been shown that 800 mg ibuprofen reduces postoperative pain and morphine consumption more effectively [16]. Unlike our study, in both these studies the first ibuprofen dose was given during wound closure and drug was administered every 6 hours in postoperative period. However, in our study, effect of addition of single dose i.v. ibuprofen to preoperative pregabalin was examined. Therefore, VAS scores were significantly lower in PACU, at the 1st and 2nd hours, whereas no statistical significance was found between groups in the 4th, 8th, and 12th hours even though VAS values were found to be lower in Group PI.

Total morphine consumption continued to be significantly lower during the first 12 hours. Results of this study show preoperative use of drugs that have different mechanisms of action and multimodal analgesia, reducing postoperative pain and morphine consumption [16]. Decreased amount of analgesic consumption in multimodal analgesia reduces incidence of undesirable side effects and increases patient comfort. Use of pregabalin together with NSAID as preemptive multimodal analgesia in lumbar surgeries has been shown to be effective in recent studies. Kim et al. reported that a multimodal preemptive regimen of celecoxib, pregabalin, oxycodone, and acetaminophen provides more effective postoperative pain control compared to intravenous morphine alone in patients having lumbar fusion surgery [17]. Garcia et al. also reported that preemptive celecoxib and pregabalin reduced both postoperative pain levels and morphine consumption in a similar study [18].

Despite its popularity in the last two decades, there are increasing concerns about studies for preventive analgesia. Preventive analgesia is demonstrated when postoperative pain or analgesic use reduced beyond the duration of the target drug, which is defined as 5.5 half-lives of the target drug [19]. When we examined our results, it was shown that pain intensity reduced only in the first 2 hours after 2-hour average surgery duration. We know that 800 mg i.v. ibuprofen has 2.4-hour elimination half-life, so we can think our analgesic effect is a direct analgesic effect in the form of multimodal analgesia with pregabalin instead of preventive analgesia [12].

Although there are reports about pregabalin reducing postoperative pain if used preoperatively, its exact mechanism of action is not clear. Even though gabapentinoids bind to alpha2kappa subunit of presynaptic voltage gated calcium channels, because this subunit's upregulation after injury takes several days, this is not thought to be the mechanism responsible for acute postoperative analgesia [20]. There are studies that report that this effect of gabapentinoids can be through noradrenergic descending pathway [21]. According to results of our study analgesic effect is increased together with the aforementioned mechanisms of NSAID, regardless of their mechanism of action.

In our study even though the amount of hemorrhage appears to be higher in ibuprofen group, there was not a statistically significant increase in hemorrhage; also there was not an increase in the need for blood transfusion. Ibuprofen, like other NSAIDs, is known to inhibit thromboxane in a dose related manner [22]. From the two drugs that are approved to be used intravenously ketorolac is contraindicated before surgery whereas i.v. ibuprofen is approved in adults for mild pain and as an adjunct to opioids for moderate to severe pain, it can be used perioperatively for surgeries other than CABG [12]. In a study with 185 orthopedic patients first dose of ibuprofen was given before anesthesia induction and even though the amount of intraoperative hemorrhage was not recorded the amount of total blood transfusions was not increased significantly [23]. In addition, it was reported that ibuprofen can be safely used regarding other side effects. We also did not see any significant increase in side effects regarding our use of single dose ibuprofen.

There are some limitations to this study. Since we wanted to evaluate preoperative effect of ibuprofen we decided to administer a single dose but postoperative analgesia levels could be higher if postoperative doses were added. Future studies should evaluate incidence of gastrointestinal and renal complications in postoperative period as a result of proposed treatment. In addition, the study would be more significant if a third group with ibuprofen given at the end of the operation was implemented and we could evaluate whether the effect of ibuprofen is preventive or not.

## 5. Conclusion

In conclusion, addition of preoperative single dose of i.v. ibuprofen to preoperative single dose of oral pregabalin in posterior lumbar interbody fusion surgery patients provided significant analgesic effect in early postoperative period and also significantly reduced postoperative morphine consumption without increasing incidence of side effects. Higher quality of analgesia can be achieved with the recommended doses of 800 mg every 6 hours, but probably increase in incidence of side effects would also be inevitable.

## Figures and Tables

**Table 1:** Demographic data of patients.

	Group Pregabalin	Group Pregabalin + Ibuprofen	*P* value
ASA (I/II)	6/15	7/14	*P* = 0.739 Chi-square: 0.111
Gender (male/female)	11/10	8/13	*P* = 0.352 Chi-square: 0.865
Age	56.4 ± 15.63	56.3 ± 10.80	*P* = 0.649 *T*: 0.459
Height	170.09 ± 8.23	165.42 ± 7.20	*P* = 0.058 *T*: −1.953
Body weight	83.61 ± 16.20	78.52 ± 9.64	*P* = 0.461 *Z*: −0.737
Body mass index	28.60 ± 3.51	28.65 ± 3.33	*P* = 0.957 *T*: 0.054

Data are shown as patient number or value ± standard deviation.

**Table 2:** Surgery related data.

	Group Pregabalin	Group Pregabalin + Ibuprofen	*P* value
Number of operated lumbar regions (1/2/3)	3/16/2	4/17/0	*P* = 0.337 Chi-square: 2.173
Duration of surgery (min)	121 ± 35.7	115.6 ± 22.3	*P* = 0.558 *T*: 0.590
Hemorrhage during surgery (ml)	264.2 ± 119.5	416.6 ± 275.3	*P* = 0.649 *T*: 0.459
Blood transfusion (yes/no)	1/20	3/18	*P* = 0.293 Chi-square: 1.105
Amount of fluids given during surgery (ml)	1700 ± 433.5	1823.8 ± 538.1	*P* = 0.085 *Z*: −1.724

Data are shown as patient number or value ± standard deviation.

**Table 3:** Adverse side effects.

	Group Pregabalin	Group pregabalin + Ibuprofen	*P* value
Nausea/vomiting (yes/no)	2/19	1/20	*P* = 0.549 Chi-square: 0.359
Sedation (yes/no)	6/15	8/13	*P* = 0.513 Chi-square: 0.429
Visual disturbances (yes/no)	2/19	1/20	*P* = 0.549 Chi-square: 0.359
Dizziness (yes/no)	6/15	8/13	*P* = 0.513 Chi-square: 0.429

Data are shown as patient number.
